# Radiological Features of Gastrointestinal Lymphoma

**DOI:** 10.1155/2016/2498143

**Published:** 2015-12-24

**Authors:** Giuseppe Lo Re, Vernuccio Federica, Federico Midiri, Dario Picone, Giuseppe La Tona, Massimo Galia, Antonio Lo Casto, Roberto Lagalla, Massimo Midiri

**Affiliations:** Radiology Section, DIBIMED, University of Palermo, Via del Vespro 129, 90127 Palermo, Italy

## Abstract

Gastrointestinal lymphomas represent 5–20% of extranodal lymphomas and mainly occur in the stomach and small intestine. Clinical findings are not specific, thus often determining a delay in the diagnosis. Imaging features at conventional and cross-sectional imaging must be known by the radiologist since he/she plays a pivotal role in the diagnosis and disease assessment, thus assisting in the choice of the optimal treatment to patients. This review focuses on the wide variety of imaging presentation of esophageal, gastric, and small and large bowel lymphoma presenting their main imaging appearances at conventional and cross-sectional imaging, mainly focusing on computed tomography and magnetic resonance, helping in the choice of the best imaging technique for the disease characterization and assessment and the recognition of potential complications.

## 1. Introduction

Gastrointestinal (GI) lymphoma accounts for 5–20% of extranodal lymphomas [1, 2]: the stomach is the most common site, followed by small intestine (ileum (60–65%), jejunum (20%−25%), and duodenum (6%–8%) and then colorectal lymphomas (6–12%)) [3–8].

GI lymphomas most commonly occur around the sixth decade of life and, although rare in childhood, they are the most common GI tumours in this age [9, 10].

Etiology is usually unknown although the increase of the incidence of non-Hodgkin lymphoma has been related to the increase of congenital and acquired immunodeficiency [1, 11].

Risk factors implicated in the pathogenesis of GI lymphoma are some infections due to* Helicobacter pylori*, human immunodeficiency virus infection,* Campylobacter jejuni*, Epstein-Barr virus, hepatitis B virus, human T-cell lymphotropic virus-1, and some inflammatory conditions as celiac disease, inflammatory bowel disease, atrophic gastritis, and parasitic infection [2, 9].

Clinical findings are not specific and this causes a delay in the diagnosis. The most common symptoms are epigastric pain, weight loss, and anorexia; nausea and vomiting in case of gastric lymphoma is uncommon, except in the later stage of the disease [9]. Other symptoms encountered in these patients are GI bleeding and the presence of an abdominal mass and bowel perforation, mainly in the small bowel [9].

Concerning the histological diagnosis, the appearance of GI lymphomas is an accumulation of lymphocytic tumour cells with a uniform pattern with an admixture of mature and immature elements [9].

The majority of GI lymphomas are of B-cell origin, while just 8%–10% show a T-cell origin [9]. Most low-grade B-cell GI lymphomas are of mucosa-associated lymphoid tissue (MALT) type, while enteropathy-associated T-cell lymphoma is the most common primary gastrointestinal T-cell lymphoma. GI lymphomas represent a heterogeneous group of entities originating from different cell lineage, with lymphoid cell at different stage of development, and with different biologic behaviour [10]. Certain histological subtypes most commonly occur in a precise location as MALT lymphoma in stomach, mantle cell lymphoma in terminal ileum, jejunum, and colon, enteropathy-associated T-cell lymphoma in jejunum, and follicular lymphoma in duodenum [2].

After a diagnosis of GI lymphoma is confirmed, the extent of disease has to be determined. Laboratory studies should include a complete blood count, HIV, HBV, and HCV serology, and liver and renal function blood tests and electrolytes.

Imaging plays a pivotal role both in the diagnostic phase and in the recognition of potential complications, as perforation, obstruction, and fistulas of the involved GI wall with the adjacent structures [12].

GI lymphoma has a wide variety of morphological imaging features at conventional and cross-sectional imaging. Primary GI lymphomas are best classified according to the classification of the Consensus Conference in Lugano in 1993 [13]: stage I is defined when the tumour is confined to GI tract, while in stage II, the most common one, the tumour is extended into the abdominal cavity with nodal involvement that can be either local (II1) or distant (II2). When the tumour penetrates through serosa involving adjacent structures, it is classified as stage III, while when a disseminated extranodal involvement or a GI tract lesion with supradiaphragmatic nodal involvement occurs it is classified as stage IV.

GI lymphoma must be differentiated from other primary GI tumours and from primary nodal lymphomas because they require different treatment management and they have a substantial different prognosis [9].

## 2. Esophageal Lymphoma

Primary* esophageal* lymphomas account for less than 1% in all primary GI lymphomas, while usually result from lymph node metastasis of the lymphomas from the cervical or mediastinal region [14]. Both findings on barium studies, as irregular filling defects, and on CT, as thickened esophageal wall with narrowed lumen, are nonspecific and mimic esophageal adenocarcinoma [14]. However, CT may be useful to differentiate primary esophageal lymphoma from lymph node involvements in the cervical or mediastinal regions, in staging of the disease and in evaluating response to therapy [14].

## 3. Gastric Lymphoma

Concerning* gastric lymphoma*, the most accepted hypothesis is that a chronic infection of the stomach by* Helicobacter pylori* causes lymphoid proliferation in the gastric mucosa, with subsequent development of gastric MALT lymphoma [5, 6]. Diffuse infiltrates of small centrocyte-like cells invading the epithelial lining of glands or crypts are the classical lymphoepithelial lesions of low-grade MALT lymphoma [15, 16]. In high-grade MALT lymphoma, confluent clusters or sheets with or without areas of low-grade component can be recognized [17]. Endoscopic ultrasonography (EUS) turns to be quite useful to demonstrate all the components of the gastric wall, the thickening of the intermediate anatomic layers (submucosa, muscularis propria), extramural infiltration, and lymph node involvement [18]. Yet, it has been proposed for evaluating the extension of gastric tumours.

Three different EUS patterns can be detected in gastric lymphomas [18]:

- Giant rigid gastric folds, sometimes determining a polypoid appearance.
- Localized or extended hypoechoic infiltration.
- Thickening with superficial stellate-shaped ulcerations.

For the differential diagnosis of lymphoma with gastric carcinomas, on EUS a more echogenic pattern and a different trend of diffusion can be demonstrated in patients with gastric carcinoma (the pattern of growth may be fungating or ulcerative and infiltrative) (no extended longitudinal hypoechoic infiltration of the superficial layers or extended hypoechoic transmural infiltration) [18].

The impact of EUS on clinical outcome is consistent, as it can predict MALT remission after the simple eradication therapy of* Helicobacter pylori* [19]. EUS is superior to CT for the staging and the assessment of the T and N parameters [6, 20]. However, compared to CT it cannot demonstrate the true extraluminal extent of the disease (M) or the involvement of distant lymph nodes.

Regarding* conventional X-ray*, the role of barium studies is limited to the detection of a lesion and to the demonstration of its location and extent.

The most common radiological signs on barium meal vary from normal to bull's eye appearance due to central ulceration, filling defects, thickened gastric mucosal folds, and linitis plastica.

Moreover, it is possible to distinguish the predominant features of early and advanced gastric lymphomas: the first usually present as shallow ulcerations or uneven mucosa with enlarging radiation folds [21]; the second are usually revealed as multiple masses or ulcerations, diffuse thickening of the folds, extensive submucosal infiltration, extension across the pylorus or the esophagogastric junction, large tumours over 10 cm in diameter, and preservation of pliability of the gastric wall due to a lack of the desmoplastic reaction [22, 23].

Though barium studies may demonstrate subtle lesions not seen at CT, they do not demonstrate the true extraluminal extent of the disease and are of little value in staging [7].

**Figure 1:**
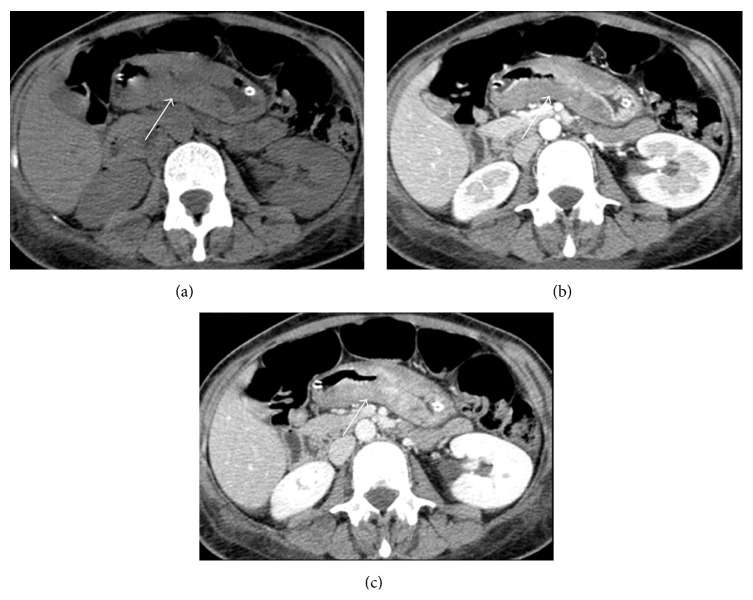
Abdominal CT scan in a 48-year-old female with gastric lymphoma. Axial pre- (a) and postcontrastographic CT scan in the arterial (b) and portal venous (c) phase show diffuse segmental (length: 9 cm) wall thickening (thickness: 1,8 cm) (arrows) of the gastric corpus and antrum with mild contrast enhancement. The patient underwent gastrectomy with ileal-jejunum anastomosis.

The most common* CT* patterns of gastric lymphoma are the presence of diffuse or segmental wall thickening of 2–5 cm with low contrast enhancement and extensive lateral extension of the tumour due to submucosal spread (Figure 1) [24]; moreover, CT can assess the presence of lymphadenopathies. Less commonly, gastric lymphoma may present on CT as a polypoidal mass, an ulcerative lesion, or a mucosal nodularity.

Considering the CT features of lymphoma, in low-grade ones there is less severe gastric wall thickening than in high-grade lymphoma, and abdominal lymphadenopathy is less common [25, 26]. The absence of abnormality or the presence of just minimal gastric wall thickening or a shallow lesion at CT suggests low-grade MALT lymphoma [26]; yet, CT is of limited value in its diagnosis [7]. A greater thickening may indicate transformation to a higher grade lymphoma [24].

Comparing EUS and CT, EUS is better in the evaluation of parietal extension of the tumour while CT better assesses the extraparietal involvement. Moreover, as previously stated, CT has several limitations in the detection of low-grade and MALT lymphomas; yet for their diagnosis and staging EUS is the best imaging technique since it can accurately assess the intramural infiltration, local node involvement, and response to therapy [27].

Comparing MR and CT, they show a similar diagnostic capability and overlapping radiological features [27], but due to high costs, long time required for each examination, and possible artifacts, MR is used just when the patient cannot be submitted to CT.

## 4. Small Bowel Lymphoma

Concerning* small bowel lymphoma*, small bowel is usually studied through endoscopic or radiological imaging techniques. Video capsule endoscopy is the preferred imaging technique for the visualization of mucosal abnormalities in patients with obscure bleeding when gastroscopy and colonoscopy are negative; however, this method is not always able to identify the source of bleeding and is contraindicated in suspected stenosis or obstruction, because of the risk of retention of the video capsule [28, 29].

Single or double balloon enteroscopy partially displays the bowel and allows biopsies; however, it is limited by its invasiveness, the long timing of the examination, and the technical difficulties [7]. Conventional radiological imaging techniques, such as the study of the small intestine through enterography or enteroclysis, allow the diagnosis of mucosal abnormalities, masses, and/or invaginations but provide only indirect information on the intestinal wall and on the surrounding structures; yet, they are actually considered obsolete [2]. CT and MR enteroclysis and enterography have an increasingly important role in the study of small intestine tumours. Thanks to their high spatial resolution, they allow a direct visualization of both the wall (assessing any luminal anomaly) and surrounding structures (mesentery, adjacent adipose tissue, lymph nodes, and peritoneal spaces) [3, 30, 31].

MR, thanks to its multiplanarity, has an excellent contrast resolution, does not use ionizing radiation, and provides both anatomical and functional information about bowel loops, allowing distinguishing organic stenosis from normal peristaltic waves [32].

**Figure 2:**
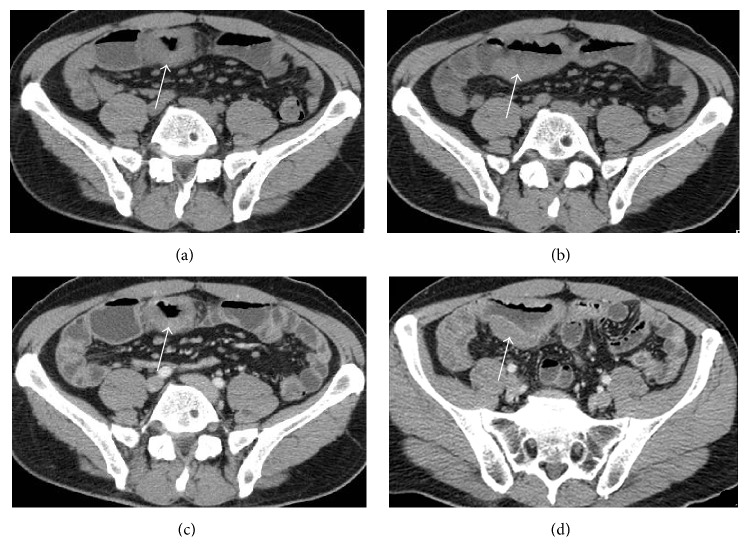
Abdominal CT scan in a 46-year-old male with celiac disease who developed an ileal lymphoma. Abdominal CT scan in the precontrastographic phase ((a) and (b)) and in the postcontrastographic phase ((c) and (d)) shows diffuse segmental (length: 8 cm) wall thickening (thickness: 1,5 cm) (arrows) with mild contrast enhancement in an ileal loop. Multiple subcentimetric lymph nodes are detected near the affected loop in the mesenteric fat.

CT is particularly useful both for staging and in the follow-up after surgery or chemoradiotherapy. Nowadays, CT allows the evaluation of wall thickness, mesenteric vasculature, and any associated extramural findings [7, 29]. Small bowel CT, or entero-CT, performed through a multislice CT scanner has led to considerable advances in the detection and staging of intestinal diseases. The advantage of this technique lies in its panoramic view, which allows the evaluation of the intestinal wall thickness, the degree of bowel distension, and the circular folds. Yet, ileal loops and also those of the deep pelvis, the mesentery, the surrounding adipose tissue, and other abdominal organs are studied (Figure 2) [20, 33].

CT-enterography is more and more used in place of conventional double contrast enteroclysis. It is performed with the patient in supine/prone position, and with craniocaudal scans, after oral administration of an isotonic solution, in order to obtain an adequate distension of small bowel wall. Less frequently, an enteroclysis CT is performed, after nasal-jejunal intubation, and subsequent introduction of a diluted barium solution. Neutral contrast media (i.e., PEG) are generally preferred for the assessment of bowel wall, particularly after intravenous contrast medium injection since the water density of the solution is opposed to that of the wall that is enhanced in the vascular phase, mainly in inflammatory diseases [20, 29, 33].

**Figure 3:**
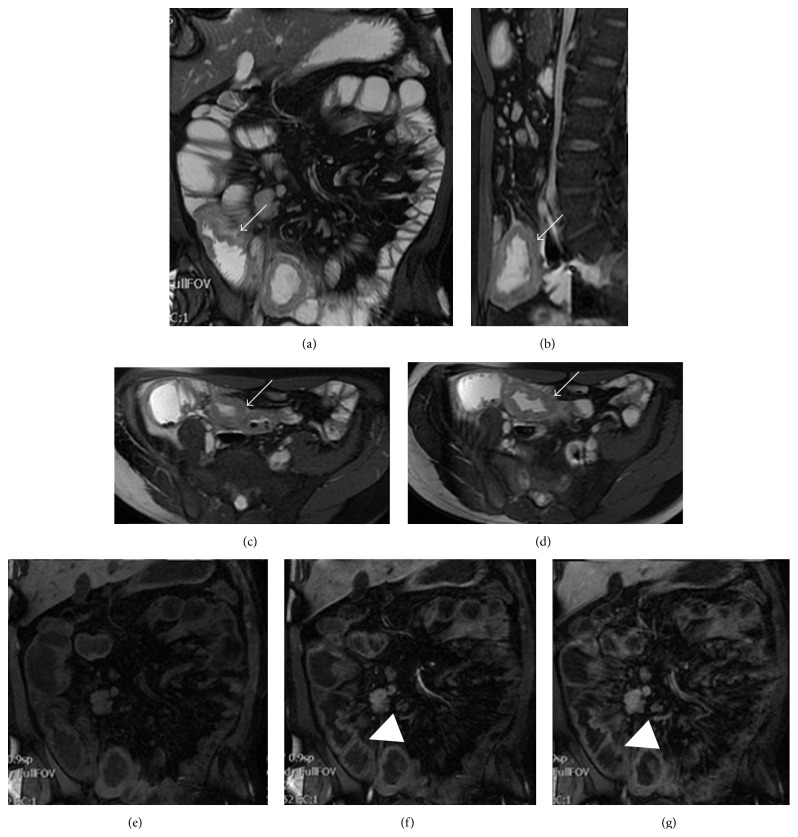
Abdominal MR enterography in the same patient of Figure 2. MR enterography shows the presence of a circumferential thickening of an ileal bowel loop (arrows) in the coronal (a), sagittal (b), and axial ((c) and (d)) planes, before contrast medium injection. Compared to the coronal precontrastographic phase (e), this thickening shows mild contrast enhancement in the arterial (f) and portal venous phase (g) (arrowheads).

MR has played a secondary role for years compared to CT, especially due to the increased length of time of acquisition and the motion artifacts [11, 34]. However, the rapid development of the technical innovations, the introduction of new equipment, and higher gradients magnetic fields has allowed the development of fast T1- and T2-weighted sequences, single-shot fast spin-echo, or gradient-echo, acquired during a single apnoea, which enabled the development of MR protocols for the study of small bowel using an intraluminal contrast medium (enterography-MR) [29]. The absence of ionizing radiation makes this method particularly suitable in the follow-up [11, 34]. At MR a diagnosis of small bowel lymphoma is suggested by the presence of an infiltrative lesion with patency of bowel lumen or a nonstenotic bowel mass, mesenteric involvement with enlarged lymph nodes, splenomegaly, and mesenteric and retroperitoneal lymphadenopathy (Figure 3) [35].

However, the imaging diagnosis of small bowel lymphoma is still based on the use of enterography CT [33, 36].

The most common CT/MR patterns of small bowel lymphoma are 5 [34, 37]:

- Polypoid/nodular pattern.
- Infiltrative pattern.
- Aneurismal pattern.
- Exophytic mass.
- Stenosing mass (rare).

The* polypoid* pattern is characterized by the presence of a solid nodule, with a homogeneous signal density/intensity, that develops in the submucosa and protrudes into the lumen appearing as a polypoid mass. There is no wall thickening and/or lymph adenopathy and the mucosa is intact. This mass may cause intussusception.

The* infiltrative* form is characterized by segmental symmetrical or slightly asymmetrical infiltrating lesions with a medium diameter of 1.5 cm and 2 cm, associated with mild circumferential thickening of the small bowel wall. Usually, the infiltrative lesions show ill-defined margins and a homogeneous contrast enhancement; the latter may rarely be inhomogeneous because of the presence of hypodense/hypointense areas due to development of necrosis and/or ischemia in the context of the lesion. These lesions may extend to the whole bowel thickness, from the endoluminal mucosa to the tunica serosa. The length of the thickened small bowel segment is variable.

**Figure 4:**
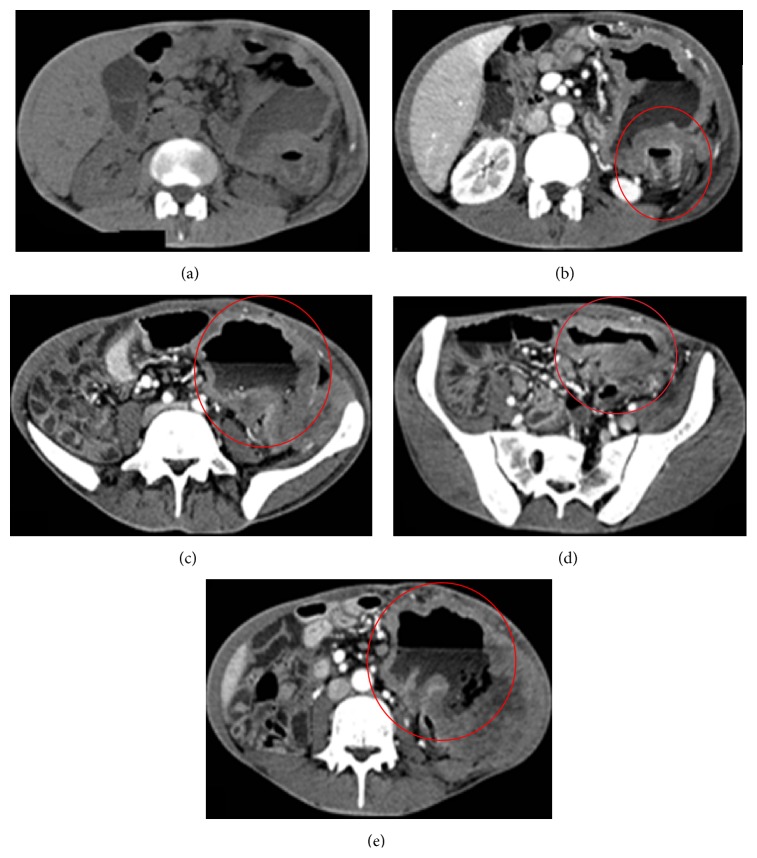
Aneurismatic jejunal lymphoma in a 43-year-old female. (a, b, c) Precontrastographic and postcontrastographic axial CT scan show severe circumferential wall thickening (thickening 17 mm), inhomogeneously hyperdense after contrast medium injection, of a jejunal ileal loop (length: 20 cm) located in the left side and left upper quadrant (red circle). Moreover, endoluminal dilatation and air-fluid level inside and enlarged lymph nodes and the surrounding mesenteric fat can be noticed (arrowheads). (d) Infiltration of the left colonic and sigmoid bowel wall (arrow). (e) Thickened bowel walls are entwined with a newly formed lymphomatous mass of the left abdominal wall that infiltrates the left abdominal wall muscles and the superior edge of the left iliac muscle.

The* aneurismal* pattern (diameter of dilatation of the lumen over 4 cm), firstly diagnosed by Cupps et al. in 1969 [4], represents 31% of small bowel lymphomas (Figure 4). It usually coexists with the infiltrative form since it can represent its natural evolution [38, 39]. Several factors are responsible for the aneurismal dilation secondary to infiltrative growth of neoplastic lesion, as a progressive destruction of myenteric plexus, destruction of muscle layers with stretching of the muscle fibers, and loss of contractile cells; on the other hand, the infiltration of arterial and lymphatic vessels determines anoxia and necrosis within the lesion. According to some authors, this tumour necrosis could lead to cavitation and be also responsible for the aneurismal dilatation [38, 39].

The* stenosing* form is a rare form of presentation of intestinal lymphoma. This pattern generally occurs in Hodgkin's lymphoma. The growth of the tumour determines concentric fibrotic stenosis of the affected loop, resulting in dislocation of the contiguous loops. It is thought that this pattern is associated with a greater fibrotic component. Compared to the stenosis occurring in other malignancies, the one observed in stenosing lymphoma determines just a minimal, if any, dilation of the upstream bowel segments, and this is due to the absence of a desmoplastic reaction.

The* mesenteric* pattern is characterized by the development of lymphoid tissue outside of the intestinal wall through the adventitia, extending in the context of nearby structures, in particular in the mesentery. In this form, lymphomas present as large exophytic masses (bulky appearance) with secondary involvement of surrounding tissues. The diameter of 70% of these tumours is at diagnosis larger than 5 cm [34, 37]. In the larger masses, larger ulcerative complications, tissue necrosis, perforation, and enteroenteric fistula formation are not uncommon [37].

Differential diagnosis includes all inflammatory, neoplastic, and metastatic lesions involving the small bowel. Primary carcinoma, metastases (especially those from melanoma and renal cancer), and the intestinal leiomyosarcoma are characterized by large necrotic/colliquative cavitations. In rare cases, inflammatory conditions, such as Crohn's disease and intestinal tuberculosis, have to be differentiated: the significant thickening of the bowel wall (greater than 2 cm), the presence of lymphomatous nodules, and the coexistence of perivisceral multiple lymph nodes are CT features that are suggestive for a lymphoproliferative process. On the other hand, discontinuous, segmental circumferential thickening (thickness of approximately 0.5–2 cm), with symmetrical and circumferential contrast enhancement, characterized by alternation of hyperdense mucosa, a submucosal hypodense halo (halo-sign), and a hyperdense outer layer, suggests inflammatory diseases [40].

## 5. Large Bowel Lymphoma

Primary lymphoma of the* large bowel* accounts for 0.4% of all tumours of the colon, and colorectal lymphomas constitute 6%–12% of gastrointestinal lymphomas [26]. The cecum and rectum are most commonly affected parts compared to other tracts of the large bowel [5].

Primary large bowel lymphoma may appear as localized, large, extraluminal masses or constricting simulating annular-type carcinomas and may present with* different radiological patterns* that are often quite similar to other large bowel tumours or inflammatory diseases, thus leading to a difficult differential diagnosis [3]. These patterns include bulky polypoidal mass, focal infiltrative tumour, and aneurismal dilatation [3].

On barium studies and on CT the most common pattern is the polypoid one: polyps may vary from few millimetres to 20 centimetres and are mainly located in the ileocecal valve. Usually, bulky lymphomatoid polypoid masses are larger than the ones that can be encountered in colorectal adenocarcinomas and may extend beyond the bowel wall, thus presenting as enormous peritoneal masses, that can also be cavitated [27].

**Figure 5:**
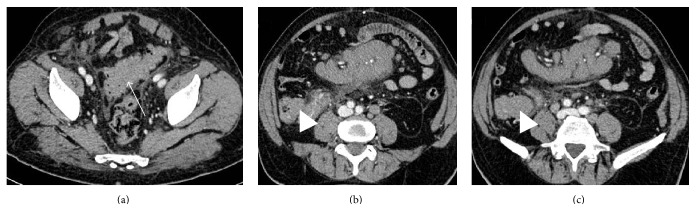
Abdominal CT scan of ileal and sigmoid lymphoma in a 78-year-old male. Axial CT scan in the portal venous phase shows a non-Hodgkin lymphoma seen as abnormal circumferential bowel thickening of the sigmoid colon ((a) arrow) and of the last ileal loop ((b) and (c) arrowheads). Enhanced lymphomatous small bowel loops (b) represent the halves of a sandwich, enveloping enhanced vessels (the sandwich filling). The patient underwent chemotherapy with marked reduction of the thickening at the follow-up CT scan.

Colorectal lymphomas may also present as a concentric circumferential bowel wall thickening (with or without ulceration) or as exophytic tumours, mucosal nodularity, and fold thickening (Figure 5) [26]. Furthermore, focal strictures, aneurismal dilatation, or ulcerative forms with fistula formation may be encountered [26]. However, some features as well-defined margins with preserved fat planes, absence of involvement of adjacent structures, and perforation without any desmoplastic response may help in the differential diagnosis of lymphoma from adenocarcinoma [7]. The latter feature is responsible for the fact that obstruction is less frequent in lymphoma compared to adenocarcinoma [7].

Colonic lymphoma usually presents with larger lesions and involves a longer segment compared to adenocarcinoma; moreover, colonic lymphoma is usually located near the ileocaecal valve and grows into the terminal ileum, not invading or obstructing neighbouring viscera [41].

However, there are no imaging findings pathognomonic for lymphoma.

GI lymphoma has a wide variety of morphological imaging features at conventional and cross-sectional imaging. The goal of the radiologist when there is a clinical suspicion of GI lymphoma is to provide a diagnosis according to the WHO classification in order to provide an optimal treatment to patients. Fiberoptic endoscopy of the GI tract has still a pivotal role in the evaluation of lymphoma occurring in the oesophagus or in the stomach; however it does not allow the evaluation of concomitant localization of the lymphoma in the GI tract, as CT does.

In patients with obscure bleeding with negative gastroscopy and colonoscopy, video capsule endoscopy is usually the preferred imaging technique for the detection of mucosal abnormalities; however, it is contraindicated to use video capsule in suspected stenosis or obstruction, because of the risk of its retention, while CT can be performed in these patients.

Moreover, while conventional imaging provides just suggestive findings of the presence of the disease, cross-sectional imaging plays an emerging role in the diagnosis and staging of GI lymphoma.

The main imaging appearance of GI lymphoma may be summarized as follows:

- Diffuse infiltrative form, which is characterized by a circumferential wall thickening of the involved GI wall, leading to destruction of the muscularis propria and autonomic plexus and subsequent dilatation of the involved segment.
- Focal GI involvement, which may appear as a solitary or multiple nodular involvement.
- Ulcerative form.

Thanks to CT it is possible to study not only the GI tract using enterography technique, but also local and distant lymph nodes and other thoracic and abdominal organs that can be affected also by the disease, thus allowing an imaging staging of the disease according to the classification of the Consensus Conference of Lugano [13].

The role of CT is also considered pivotal in the evaluation of complications of the disease, as perforation, fistulisation, and obstruction, and in the differential diagnosis with other neoplastic or inflammatory conditions, which may also coexist with the lymphoma [42].

Lastly, CT must be actually considered also the preferred technique for the evaluation of response to therapy when medical therapy with targeted therapy is used; in this case according to the used drug, the imaging appearance may be substantially different.

However, CT has still many limitations for staging, restaging, and response to therapy assessment of lymphoma [43]. To date, 2-[fluorine-18]fluoro-2-deoxy-d-glucose (FDG) positron emission tomography (PET) is considered the imaging modality of choice for staging and follow-up in Hodgkin disease and most non-Hodgkin lymphomas [44]. In particular, considering GI lymphoma, FDG uptake is different according to the different location: esophageal lymphoma manifests as circumferential thickening of the esophageal wall with increased FDG uptake; gastric lymphoma presents with a variable, usually diffuse FDG uptake that can involve all portions of the stomach and that is usually higher than the liver one; small bowel lymphoma is characterized on FDG PET/CT by the presence of multiple foci of intense radiotracer activity arranged in a curvilinear pattern; finally, large bowel lymphoma manifests with a characteristic pattern of uptake consisting of focal, nodular, or diffuse hypermetabolic activity [44]. However, normal peristaltic activity, normal gastrointestinal lymphoid tissue, and granulomatous or inflammatory conditions represent a limit of the PET/CT for the evaluation of possible lymphomatous involvement in both small and large bowel lymphoma [44].

On the other hand, MR provides a better evaluation of the bowel wall and of the local infiltration by the disease. Whole-body MR with diffusion weighted imaging proved to be useful in nodal and bone marrow staging of lymphoma [45]. Concerning GI lymphoma, diffusion weighted imaging proved to be useful in the detection of gastric lymphoma, since the latter shows an increased signal on diffusion weighted imaging sequence and decreased signal on apparent diffusion coefficient maps, and in the differentiation from adenocarcinoma with significantly lower apparent diffusion coefficient values of adenocarcinoma compared to lymphoma [46]. According to our opinion, also for small bowel lymphoma, diffusion weighted imaging may help in its detection. However, MR study is mainly focused on the evaluation of the gastrointestinal tract, not allowing an accurate evaluation of the thoracic and other abdominal organs.

However, although findings of the different imaging techniques may be suspicious for lymphoma, tissue biopsy is always necessary for a specific diagnosis.

## References

[1] Bäck H., Gustavson B., Ridell B., Rödjer S., Westin J. (1986). Primary gastrointestinal lymphoma incidence, clinical presentation, and surgical approach. *Journal of Surgical Oncology*.

[2] Ghimire P., Wu G.-Y., Zhu L. (2011). Primary gastrointestinal lymphoma. *World Journal of Gastroenterology*.

[3] Levine M. S., Rubesin S. E., Pantongrag-Brown L., Buck J. L., Herlinger H. (1997). Non-Hodgkin's lymphoma of the gastrointestinal tract: radiographic findings. *American Journal of Roentgenology*.

[4] Cupps R. E., Hodgson J. R., Dockerty M. B., Adson M. A. (1969). Primary lymphoma in the small intestine: problems of roentgenologic diagnosis. *Radiology*.

[5] Van de Water J. M. W., Cillessen S. A. G. M., Visser O. J., Verbeek W. H. M., Meijer C. J. L. M., Mulder C. J. J. (2010). Enteropathy associated T-cell lymphoma and its precursor lesions. *Best Practice and Research: Clinical Gastroenterology*.

[6] Krol A. D. G., le Cessie S., Snijder S., Kluin-Nelemans J. C., Kluin P. M., Noordijk E. M. (2003). Primary extranodal non-Hodgkin's lymphoma (NHL): the impact of alternative definitions tested in the Comprehensive Cancer Centre West population-based NHL registry. *Annals of Oncology*.

[7] Ghai S., Pattison J., Ghai S., O'Malley M. E., Khalili K., Stephens M. (2007). Primary gastrointestinal lymphoma: spectrum of imaging findings with pathologic correlation. *Radiographics*.

[8] Masselli G., Polettini E., Casciani E., Bertini L., Vecchioli A., Gualdi G. (2009). Small-bowel neoplasms: prospective evaluation of MR enteroclysis. *Radiology*.

[9] Smith C., Kubicka R. A., Thomas C. R. (1992). Non-Hodgkin lymphoma of the gastrointestinal tract. *RadioGraphics*.

[10] Lewis R. B., Mehrotra A. K., Rodríguez P., Manning M. A., Levine M. S. (2014). From the radiologic pathology archives: gastrointestinal lymphoma: radiologic and pathologic findings. *RadioGraphics*.

[11] Laghi A., Passariello R. (2003). La risonanza magnetica nello studio del piccolo intestino. *La Radiologia Medica*.

[12] Sandrasegaran K., Rajesh A., Rydberg J., Rushing D. A., Akisik F. M., Henley J. D. (2005). Gastrointestinal stromal tumors: clinical, radiologic, and pathologic features. *American Journal of Roentgenology*.

[13] Rohatiner A., d'Amore F., Coiffier B., et al (1994). Report on a workshop convened to discuss the pathological and staging classifications of gastrointestinal tract lymphoma. *Annals of Oncology*.

[14] Peng J. C., Zhong L., Ran Z. H. (2015). Primary lymphomas in the gastrointestinal tract. *Journal of Digestive Diseases*.

[15] Isaacson P. G., Wright D. H. (1984). Extranodal malignant lymphoma arising from mucosa-associated lymphoid tissue. *Cancer*.

[16] Isaacson P. G., Spencer J., Finn T. (1986). Primary B-cell gastric lymphoma. *Human Pathology*.

[17] Isaacson P. G. (1994). Gastrointestinal lymphoma. *Human Pathology*.

[18] Bolondi L., Casanova P., Caletti G. C., Grigioni W., Zani L., Barbara L. (1987). Primary gastric lymphoma versus gastric carcinoma: endoscopic US evaluation. *Radiology*.

[19] Caletti G., Zinzani P. L., Fusaroli P. (2002). The importance of endoscopic ultrasonography in the management of low-grade gastric mucosa-associated lymphoid tissue lymphoma. *Alimentary Pharmacology & Therapeutics*.

[20] Balthazar E. J., Noordhoorn M., Megibow A. J., Gordon R. B. (1997). CT of small-bowel lymphoma in immunocompetent patients and patients with AIDS: comparison of findings. *American Journal of Roentgenology*.

[21] Sato T., Sakai Y., Ishiguro S., Furukawa H. (1986). Radiologic manifestations of early gastric lymphoma. *American Journal of Roentgenology*.

[22] Menuck L. S. (1976). Gastric lymphoma, a radiologic diagnosis. *Gastrointestinal Radiology*.

[23] Hricak H., Thoeni R. F., Margulis A. R., Eyler W. R., Francis I. R. (1980). Extension of gastric lymphoma into the esophagus and duodenum. *Radiology*.

[24] Buy J. N., Moss A. A. (1982). Computed tomography of gastric lymphoma. *American Journal of Roentgenology*.

[25] Park M.-S., Kim K. W., Yu J.-S. (2002). Radiographic findings of primary B-cell lymphoma of the stomach: low-grade versus high-grade malignancy in relation to the mucosa-associated lymphoid tissue concept. *American Journal of Roentgenology*.

[26] Choi D., Lim H. K., Lee S. J. (2002). Gastric mucosa-associated lymphoid tissue lymphoma: helical CT findings and pathologic correlation. *American Journal of Roentgenology*.

[27] Guglielmi G., Schiavon F., Cammarota T. (2006). *Radiologia geriatrica*.

[28] Maglinte D. D. T. (2005). Capsule imaging and the role of radiology in the investigation of diseases of the small bowel. *Radiology*.

[29] Hara A. K., Leighton J. A., Sharma V. K., Heigh R. I., Fleischer D. E. (2005). Imaging of small bowel disease: comparison of capsule endoscopy, standard endoscopy, barium examination, and CT. *Radiographics*.

[30] Dawson I. M., Cornes J. S., Morson B. C. (1961). Primary malignant lymphoid tumors of the intestinal tract. Report of 37 cases with a study of factors influencing prognosis. *British Journal of Surgery*.

[31] Swerdlow S. H., Campo E., Harris N. L. (2008). *WHO Classification of Tumours of Haematopoietic and Lymphoid Tissues*.

[32] Heye T., Stein D., Antolovic D., Dueck M., Kauczor H.-U., Hosch W. (2012). Evaluation of bowel peristalsis by dynamic cine MRI: detection of relevant functional disturbances—initial experience. *Journal of Magnetic Resonance Imaging*.

[33] Minordi L. M., Vecchioli A., Mirk P., Filigrana E., Poloni G., Bonomo L. (2007). Multidetector CT in small-bowel neoplasms. *Radiologia Medica*.

[34] Crusco F., Pugliese F., Maselli A. (2010). Malignant small-bowel neoplasms: spectrum of disease on MR imaging. *Radiologia Medica*.

[35] Masselli G., Colaiacomo M. C., Marcelli G. (2012). MRI of the small-bowel: how to differentiate primary neoplasms and mimickers. *British Journal of Radiology*.

[36] Buckley J. A., Fishman E. K. (1998). CT evaluation of small bowel neoplasms: spectrum of disease. *Radiographics*.

[37] Smith C., Kubicka R. A., Thomas C. R. (1992). Non-hodgkin lymphoma of the gastrointestinal tract. *Radiographics*.

[38] Norfray J., Calenoff L., Zanon B. (1973). Aneurysmal lymphoma of the small intestine. *American Journal of Roentgenology*.

[39] Maizlin Z. V., Brown J. A., Buckley M. R. E. (2006). Case of the season: aneurysmal dilatation of the small bowel (not only lymphoma). *Seminars in Roentgenology*.

[40] Lo Re G., Cappello M., Tudisca C. (2014). CT enterography as a powerful tool for the evaluation of inflammatory activity in Crohn's disease: relationship of CT findings with CDAI and acute-phase reactants. *Radiologia Medica*.

[41] Beaton C., Davies M., Beynon J. (2012). The management of primary small bowel and colon lymphoma—a review. *International Journal of Colorectal Disease*.

[42] Galia M., Agnello F., La Grutta L. (2015). Computed tomography of bowel obstruction: tricks of the trade. *Expert Review of Gastroenterology & Hepatology*.

[43] Kostakoglu L., Goldsmith S. J. (2000). Fluorine-18 fluorodeoxyglucose positron emission tomography in the staging and follow-up of lymphoma: is it time to shift gears?. *European Journal of Nuclear Medicine*.

[44] Paes F. M., Kalkanis D. G., Sideras P. A., Serafini A. N. (2010). FDG PET/CT of extranodal involvement in non-Hodgkin lymphoma and Hodgkin disease. *Radiographics*.

[45] Gu J., Chan T., Zhang J., Leung A. Y. H., Kwong Y.-L., Khong P.-L. (2011). Whole-body diffusion-weighted imaging: the added value to whole-body MRI at initial diagnosis of lymphoma. *American Journal of Roentgenology*.

[46] Avcu S., Arslan H., Unal O., Kotan C., Izmirli M. (2012). The role of diffusion-weighted MR imaging and ADC values in the diagnosis of gastric tumors. *JBR-BTR*.

